# Operative Surgery

**Published:** 1887-03

**Authors:** 


### Book Reviews.

OPERATIVE SURGERY. By Stephen Smith, M. D. Lea Brothers & Co., Philadelphia. 1887.

